# Serum uric acid did not affect embryonic and pregnancy outcomes in women without PCOS during IVF procedures

**DOI:** 10.3389/fendo.2024.1310122

**Published:** 2024-02-16

**Authors:** Niwei Yan, Junli Song, Huiying Jie, Pingyin Lee, Simin Liu, Yuan Yuan

**Affiliations:** ^1^ Reproductive Medicine Center, The First Affiliated Hospital, Sun Yat-sen University, Guangzhou, China; ^2^ Guangdong Provincial Key Laboratory of Reproductive Medicine, Guangzhou, China; ^3^ Department of Surgery, Li Ka-shing Faculty of Medicine, The University of Hong Kong, Hong Kong, Hong Kong SAR, China

**Keywords:** serum uric acid, IVF treatment, mature oocyte rate, reproductive outcomes, perinatal outcomes

## Abstract

**Objectives:**

Serum uric acid (UA) levels are associated with many systemic diseases. A previous study confirmed the association between high serum uric acid levels and poor prognosis of *in vitro* fertilization (IVF) treatment in polycystic ovary syndrome (PCOS) patients. This study aimed to explore the correlation between serum uric acid levels and reproductive outcomes in patients without PCOS.

**Methods:**

A retrospective study that included 1057 patients who underwent pre-implantation genetic testing for monogenic disorders (PGT-M) treatment from January 2013 to December 2020 was conducted. The study population was further divided into 3 groups according to serum UA levels: the ≤250 μmol/L group, the 251-360 μmol/L group, and the >360 μmol/L group. The controlled ovarian hyperstimulation (COH) treatment outcomes, embryonic treatment outcomes and pregnancy outcomes of the first frozen embryo transfer (FET) cycle were compared among groups. Multivariable linear regression and binary regression were applied to detect the association between IVF outcomes and serum uric acid levels.

**Results:**

The number of retrieved oocytes, fertilization rate, viable embryo rate, blastocyst formation rate and euploid rate were not associated with serum uric acid levels. The mature oocyte rate was negatively correlated with serum uric acid levels. The pregnancy outcomes of the first FET cycle were also not associated with serum uric acid levels. After adjustment for BMI, the perinatal outcomes were not associated with serum uric acid levels.

**Conclusion:**

IVF treatment outcomes were not associated with serum uric acid levels in patients without PCOS.

## Introduction

Uric acid (UA) is the end catabolic product of purine metabolism. It has both pro-oxidant and antioxidant properties and plays a central role as a scavenger of free radicals (1, 2). A previous study revealed that UA could trigger systemic peripheral inflammation (3). UA also acts as a protective antioxidant (4). As a result, both hyperuricemia and hypouricemia are associated with many diseases, such as renal insufficiency, cardiovascular events and pulmonary comorbidities (5).

Several studies have revealed that UA accumulation may cause reproductive disorders. Elevated UA levels could inhibit nitric oxide production, which becomes a proinflammatory factor and impairs endothelial repair. UA is used as a biomarker for placental ischemia (6). Due to dysregulation of trophoblast invasion and inefficiency of physiological transformation of spiral arteries (7–9), elevated UA levels not only compromise placental vasculature but also affect maternal vasculature (10, 11). Therefore, high serum UA levels are a risk factor for many pregnancy complications (11–13).

Serum UA levels depend on the balance of production (both exogenous and endogenous) and excretion (urine or feces), which correlates strongly with metabolic status. Some metabolic disorders, such as diabetes mellitus, insulin resistance and obesity, are associated with high serum UA levels (14–16). Among infertile females, patients with polycystic ovarian syndrome (PCOS) are at high risk of metabolic syndrome. A recent study reported that elevated serum UA levels are associated with decreased probabilities of live birth and clinical pregnancy and an increased risk for low birthweight in women with PCOS (17). Because of the special characteristics of patients with PCOS, we could not apply this to the general population. To the best of our knowledge, no study has yet investigated the relationships between serum UA and reproductive outcomes of *in vitro* fertilization, except in patients with PCOS. Most of the available data have focused on pregnancy complications (11–13), and less is known about the effect of UA on the quality of oocytes or embryos.

Therefore, we conducted a study to explore the association between serum UA levels and reproductive outcomes among a non-PCOS population undergoing preimplantation genetic testing for monogenic disorders (PGT-M).

## Methods

### Study population

This retrospective study recruited patients who underwent PGT-M treatment in the Reproductive Medicine Center of the First Affiliated Hospital of SUN Yat-sen University from January 2013 to December 2020. Written consent was obtained from each participant, and this study was approved by the Ethics Committee of the First Affiliated Hospital of SUN Yat-sen University.

The inclusion criteria of this study were as follows: 1. patients underwent PGT-M treatment with aneuploid screening and obtained at least 1 euploid embryo; 2. demographic characteristics, including serum uric acid levels, were available; and 3. patient age ranged from 22 to 38.

The exclusion criteria of this study were as follows: 1. PCOS diagnosis based on the Rotterdam Criteria; 2. systematic disease (liver, renal, cardiovascular and pulmonary system); 3. underwent medication for hyperuricemia; and 4. underwent medication that would interfere with the glomerular filtration rate.

### Uric acid level

All the patients’ fasting peripheral blood samples were collected before controlled ovarian hyperstimulation (COH). All the samples were assayed using the same uricase-based colorimetric protocol from Pointe Scientific (Canton, MI) Kit U7581-120 with the lowest detection limit of 88.4 μmol/L in the central laboratory of the First Affiliated Hospital of SUN Yat-sen University. The variance coefficient was 9.0%. The presence of more than 360 μmol/L serum UA was considered hyperuricemia according to our laboratory standard referring to the study on Chinese population for women’s diagnostic standard (18).

### Clinical treatment procedures

Routine COH protocols were implemented, including an agonist long protocol, an antagonist protocol and mild ovarian stimulation cycles. Both recombined and highly purified urinary gonadotropins were utilized. Final oocyte maturation was typically induced with 6,000 to 10,000 IU of human chorionic gonadotropin (hCG) when at least three follicles had reached a maximal diameter of 18 mm. Transvaginal oocyte retrieval was performed 36 hours after human chorionic gonadotropin administration.

### Embryonic laboratory procedures

The intracytoplasmic sperm injection, embryo culture, and blastocyst culture procedures were routine procedures. The assessment method of embryo viability was morphological grading. Cleavage-stage embryos were graded according to the Istanbul consensus (19). Blastocyst quality assessment was based on the scoring system of Gardner and Schoolcraft (20). Usable blastocysts were biopsied on Days 5 or 6. Monogenetic disease was diagnosed with specific probes. Aneuploid screening was performed through next-generation sequencing (NGS) or a single-nucleotide polymorphism (SNP) microarray platform. All usable euploid blastocysts were cryopreserved for future use. The detailed vitrification and thawing procedures were as reported in a previous study (21).

### Follow-up

The first frozen embryo transfer cycle was followed. The early pregnancy outcomes were obtained by clinical visits, including pregnancy rate, clinical pregnancy rate, ectopic pregnancy rate and miscarriage rate. The perinatal outcomes were obtained through telephone interviews, including late miscarriage, live birth, newborn birthweight, maternal complications (gestational hypertension disease, gestational diabetes mellitus, premature membrane rupture, macrosomia, placenta previa, placenta accreta, polyhydramnios, and oligohydramnios) and fetal deformity. The definitions of these outcomes were the same as those in our previously reported study (22).

### Statistical analysis

Continuous data are presented as the mean ± standard deviation, and one-way analysis of variance (ANOVA) was performed for intergroup comparisons, Bonferroni corrections were applied when needed. Categorical data are presented as percentages, and the chi-square test was used for intergroup comparisons. Associations between serum UA levels and embryonic treatment outcomes were assessed using multivariable linear regression with the backward method. The association between serum UA levels and pregnancy outcomes was assessed by logistic regression. The results were considered to be statistically significant when the P value was less than 0.05. All analyses were performed using SPSS version 26 (IBM).

## Results

A total of 1219 patients aged 22 to 38 years who underwent PGT-M treatment were screened. A total of 162 patient records were excluded from the analysis: 41 for no basal serum UA level, 77 for PCOS diagnosis, 12 for liver or renal disease, and 32 for medicine usage. Finally, 1057 patients were included and further divided into 3 groups for further analysis according to their basal serum UA levels: the ≤250 μmol/L group (N=347), the 251-360 μmol/L group (N=558), and the >360 μmol/L group (N=152).

The demographic data of the three groups are listed in **Table 1**. Body mass index (BMI) values were significantly different between the groups and increased with increasing levels of basal serum UA. The other baseline parameters were comparable between groups.

**Table 1:** Baseline characteristics of the study population.

Parameter	≤250 μmol/L	251-360 μmol/L	>360 μmol/L	P	P_1_	P_2_	P_3_
Age (years)	31.65 ± 4.64	31.54 ± 4.24	31.47 ± 4.51	0.682	0.733	0.621	0.843
BMI (kg/m2)	20.45 ± 2.35	22.42 ± 2.23	22.83 ± 2.31	0.000	0.000	0.000	0.000
AMH (ng/mL)	3.72 ± 2.71	4.07 ± 3.22	3.89 ± 2.67	0.434	0.126	0.582	0.512
FSH (IU/L)	5.87 ± 2.08	5.73 ± 1.88	5.60 ± 1.74	0.386	0.468	0.275	0.446
LH (IU/L)	3.60 ± 1.98	3.51 ± 2.32	3.38 ± 2.14	0.402	0.532	0.253	0.331
E_2_ (pg/mL)	35.95 ± 20.39	34.12 ± 19.43	34.52 ± 20.12	0.711	0.423	0.721	0.792
PRL (uIU/mL)	16.69 ± 12.40	16.72 ± 12.75	16.15 ± 16.42	0.824	0.985	0.793	0.638
T (pmol/L)	0.45 ± 2.50	0.40 ± 2.03	0.38 ± 1.07	0.793	0.727	0.603	0.975

BMI, body mass index; AMH, anti-Mullerian hormone; FSH, follicle-stimulating hormone; LH, luteinizing hormone; E_2_, estradiol; PRL, prolactin; T, testosterone. P_1_, P value between ≤250 μmol/L group and 251-360 μmol/L group; P_2_, P value between ≤250 μmol/L group and >360 μmol/L group; P_3_, P value between 251-360 μmol/L group and >360 μmol/L group. The data are expressed as the mean ± SD, and the data of the three groups were analyzed by one-way analysis of variance (ANOVA). P<0.05 is considered to indicate a significant difference.

The proportion of COH protocols was comparable among the three groups. The starting stimulation dosage, stimulation duration, total gonadotropin usage, estradiol (E_2_) level on the day of hCG administration and number of oocytes retrieved among the three groups were equivalent. However, follicle stimulating hormone (FSH) levels on the day of hCG administration in the <250 μmol/L group were significantly higher than those in the other two groups. Luteinizing hormone (LH) levels on the day of hCG administration in the >360 μmol/L group were significantly higher than those in the other two groups. The proportion of pre-implantation testing (PGT) platforms was comparable among the three groups. The details are provided in **Table 2**.

**Table 2:** Clinical treatment characteristics of the study population.

Parameter	≤250 μmol/L	251-360 μmol/L	>360 μmol/L	P	P_1_	P_2_	P_3_
Protocol	–	–	–	0.664	0.563	0.627	0.832
Agonist	70.61% (245)	67.03% (374)	65.78% (100)	–	–		
Antagonist	28.24% (98)	31.90% (178)	34.21% (52)	–	–		
Mild stimulation	1.15% (4)	1.07% (6)	0.00% (0)	–	–	–	
Starting GN dosage (IU)	220.57 ± 63.45	223.52 ± 64.37	225.33 ± 61.74	0.643	0.684	0310	0.736
Total GN dosage (IU)	2385.55 ± 869.95	2421.64 ± 892.49	2484.34 ± 907.27	0.392	0.527	0.218	0.473
COS duration (day)	10.43 ± 1.83	10.33 ± 1.91	10.44 ± 1.96	0.633	0.423	0.962	0.372
FSH on HCG Day (IU/L)	14.52 ± 5.44	13.44 ± 5.42	13.33 ± 5.22	0.548	0.047	0.031	0.921
LH on HCG Day (IU/L)	1.13 ± 1.17	1.27 ± 1.32	1.59 ± 1.74	0.275	0.335	0.002	0.022
E_2_ on HCG Day (pg/mL)	3023.26 ± 1363.79	3014.21 ± 1368.95	2947.55 ± 1476.63	0.752	0.913	0.593	0.627
P on HCG Day (ng/mL)	0.91 ± 1.15	0.86 ± 0.64	0.82 ± 0.52	0.489	0.264	0.174	0.855
No. of oocytes	19.00 ± 9.13	19.32 ± 9.76	18.78 ± 9.43	0.715	0.760	0.843	0.542
PGT platform				0.492	0.541	0.248	0.762
NGS	67.44% (234)	69.89% (390)	72.37% (110)				
SNP	32.56% (113)	30.11% (168)	27.63% (42)				

IU, international unit; GN, gonadotropins; COS, controlled ovarian stimulation; FSH, follicle-stimulating hormone; HCG, human chorionic gonadotropin; LU, luteinizing hormone; E_2_, estradiol; p, progesterone; No, number; PGT-A, preimplantation genetic testing for aneuploidies; NGS, next-generation sequencing; SNP, single-nucleotide polymorphism; P_1_, P value between ≤250 μmol/L group and 251-360 μmol/L group; P_2_, P value between ≤250 μmol/L group and >360 μmol/L group; P_3_, P value between 251-360 μmol/L group and >360 μmol/L group. Continuous data are presented as the mean ± standard deviation, and one-way analysis of variance (ANOVA) was performed for intergroup comparisons. Categorical data are presented as percentages, and the chi-square test was used for intergroup comparisons. P<0.05 is considered to indicate a significant difference.

The embryonic parameters, such as mature oocyte rate, fertilization rate, multipronuclear (MPN) rate, cleavage rate, viable embryo rate, blastocyst formation rate, and euploid rate, were all comparable among groups. The details are presented in **Table 3**.

**Table 3:** Embryonic outcomes of the study population.

Parameter	≤250 μmol/L	251-360 μmol/L	>360 μmol/L	P	P_1_	P_2_	P_3_
Mature oocyte rate	0.834 ± 0.138	0.823 ± 0.136	0.821 ± 0.134	0.465	0.231	0.163	0.842
Fertilization rate	0.676 ± 0.165	0.659 ± 0.164	0.652 ± 0.163	0.332	0.148	0.101	0.674
MPN rate	0.015 ± 0.048	0.014 ± 0.042	0.012 ± 0.035	0.734	0.914	0.328	0.457
Cleavage rate	0.986 ± 0.039	0.987 ± 0.027	0.984 ± 0.046	0.612	0.735	0.524	0.326
Viable embryo rate	0.429 ± 0.181	0.412 ± 0.179	0.404 ± 0.176	0.349	0.175	0.112	0.544
Blastocyst formation rate	0.698 ± 0.194	0.689 ± 0.192	0.686 ± 0.197	0.672	0.522	0.517	0.823
Euploid rate	0.537 ± 0.285	0.542 ± 0.282	0.557 ± 0.291	0.538	0.882	0.282	0.438

MPN, multiple pronuclear; P_1_, P value between ≤250 μmol/L group and 251-360 μmol/L group; P_2_, P value between ≤250 μmol/L group and >360 μmol/L group; P_3_, P value between 251-360 μmol/L group and >360 μmol/L group. The data are expressed as the mean ± SD, and the data of the three groups were analyzed by one-way analysis of variance (ANOVA). P<0.05 is considered to indicate a significant difference.

The pregnancy outcomes of the first frozen embryo transfer cycle were recorded. Details are presented in **Table 4**. The maternal complication rate in the >360 μmol/L group was significantly higher than that in the 250-360 μmol/L group. The other pregnancy outcomes in the three groups were all comparable.

**Table 4:** Pregnancy outcomes of the study population.

Parameter	≤250 μmol/L	251-360 μmol/L	>360 μmol/L	P	P_1_	P_2_	P_3_
Pregnancy rate	63.69% (221/347)	67.74% (378/558)	62.50% (95/152)	0.432	0.220	0.840	0.245
Clinical pregnancy rate	57.93% (201/347)	62.01% (346/558)	57.24% (87/152)	0.558	0.235	0.922	0.303
Miscarriage rate	7.96% (16/201)	6.36% (22/346)	8.05% (7/87)	0.672	0.489	1.000	0.631
Ectopic pregnancy rate	0.99% (2/201)	0.58% (2/346)	0 (0/87)	0.841	0.627	1.000	1.000
Multiple pregnancy rate	2.49% (5/201)	1.16% (4/346)	1.15% (1/87)	0.639	0.300	0.672	1.000
Live birth rate	52.73% (183/347)	57.71% (322/558)	52.63% (80/152)	0.428	0.149	1.000	0.269
Newborn weight (g)	3323 ± 42	3326 ± 58	3376 ± 79	0.632	0.942	0.441	0.517
Gestational age (days)	269 ± 2	269 ± 1	268 ± 1	0.864	0.993	0.782	0.803
Low birth weight rate	3.72% (7/188)	2.45% (8/326)	2.47% (2/81)	0.749	0.425	0.728	1.000
Maternal complication rate	10.93% (20/183)	8.39% (27/322)	16.3% (13/80)	0.215	0.338	0.232	0.035
Fetal deformity rate	0 (0/188)	0.31% (1/326)	0 (0/81)	1.000	1.000	–	1.000

P_1_, P value between ≤250 μmol/L group and 251-360 μmol/L group; P_2_, P value between ≤250 μmol/L group and >360 μmol/L group; P_3_, P value between 251-360 μmol/L group and >360 μmol/L group. Continuous data are presented as the mean ± standard deviation, and one-way analysis of variance (ANOVA) was performed for intergroup comparisons. Categorical data are presented as percentages, and the chi-square test was used for intergroup comparisons. P<0.05 is considered to indicate a significant difference. “-“ means between 251 and 360 μmol/L.

We used multivariable linear regression to analyze the association between serum UA level and embryonic treatment outcomes and further adjusted for BMI, FSH and LH level on the hCG Day. Among all the factors, mature oocyte rate was individually negatively related to the serum UA level. The details are shown in **Table 5**.

**Table 5:** Association between serum UA level and reproductive outcomes outcomes.

Outcome variables	serum UA levels		
	β/OR	95% CI	p value
No. of oocytes	0.933 ^a^	0.892-0.978	0.241
Mature oocyte rate	0.023 ^a^	0.002-0.301	0.004
Fertilization rate	0.386 ^a^	0.012-9.749	0.548
MPN rate	0.075 ^a^	0.000-982.623	0.723
Cleavage rate	7.482 ^a^	0.001-134.835	0.665
Viable embryo rate	1.395 ^a^	0.011-232.334	0.936
Blastocyst formation rate	1.789 ^a^	0.043-93.284	0.859
Euploid rate	0.632 ^a^	0.173-2.946	0.584
Peak EM thickness of ET cycle	0.842 ^a^	0.278-2.143	0.392
Clinical pregnancy rate	2.374 ^b^	0.449-12.497	0.385
Live birth rate	0.402 ^b^	0.232-1.524	0.126
Maternal complication rate	0.957 ^b^	0.472-3.248	0.082

MPN, multiple pronuclear; EM, endometrium; ET, embryo transfer; The association between serum uric acid level and continuous variables was assessed by multivariable linear regression; and the association between serum UA levels and pregnancy outcomes was assessed by logistic regression; a for regression coefficient (β) and b for OR; The results were considered to be statistically significant when P<0;05.

## Discussion

The results of our retrospective study indicated that serum UA levels were not associated with reproductive outcomes in the non-PCOS population. After adjustment for BMI, the correlation of high serum UA levels and increased maternal complication rates was not significant. The presence of more than 360 μmol/L serum UA was considered hyperuricemia in the study hence less than or equal to 360 μmol/L serum UA was considered to be the normal among female population. Within in within the normal range of serum UA levels, serum UA level ≤250 μmol/L was reported to be associated a poorer outcome in clinical patients (23), which led us to apply 250 μmol/L as the another cut-off value in patients with normal serum UA levels.

Obesity is a metabolic disorder that is associated with high serum UA levels (24, 25). In obesity, UA can be transformed into a pro-oxidant and plays a direct role in the proliferation of fat cells, which promotes the oxidative stress response (26). Regarding reproduction, it was reported that the majority of women with obesity and PCOS had hyperuricemia, which was nearly threefold higher than that in women without PCOS or a normal BMI (27). It was also demonstrated that follicles from overweight women have increased oxidative stress and reduced antioxidant capacity compared to women with normal weight due to changes in the microenvironment caused by elevated UA levels (28–30). In our study, we observed a positive correlation between BMI and elevated serum UA levels, which was consistent with the findings that BMI was positively correlated with serum UA levels and that healthy women with high serum UA levels had a higher incidence of obesity than those with normal serum UA levels (30, 31). Although the average BMI in the 3 groups was within the normal range in our study, we still observed a positive correlation between BMI and serum UA levels. Therefore, we assumed that BMI was independently related to elevated serum UA levels and that the incidence of the oxidative stress response caused by serum UA accumulation increased with increasing BMI. To illustrate the association between serum UA levels and IVF treatment outcomes, we have to rule out this confounding factor. After adjustment for BMI, except for the mature oocyte rate, the embryonic treatment outcomes and pregnancy outcomes were not correlated with serum UA levels.

In addition to oxidative stress/antioxidant capacity, UA could change the follicular microenvironment and lead to insufficient blood supply to the follicles. Increased synthetic enzyme XOR-related reactive oxygen species (ROS) production contributes to endothelial dysfunction (2, 32). Proliferation and apoptosis of vascular smooth muscle cells create an inflammatory state (33). High levels of monosodium urate (MSU)-produced pro-IL-1-converted IL-1β trigger the systemic sterile inflammatory response (34). Theoretically, these aforementioned processes cause many setbacks to follicular development and might result in low-quality oocytes, which could further impair embryo formation. However, in our study, the number of oocytes retrieved, fertilization rate, viable embryo rate, blastocyst formation rate and euploid rate were not associated with serum UA levels. Only the mature oocyte rate decreased when the serum UA level increased. We assume that this correlation might be the result of time. Similar to other chronic systematic changes, such as high blood pressure and high blood glucose, a long time is needed to create observational damage. Oocyte development is a relatively short time-consuming process, so the harmful effect is not obvious. Although the mature oocyte rate decreased as the serum UA level increased, the overall clinical treatment outcomes were not compromised.

Recently, published research has demonstrated that an elevated serum uric acid level is associated with decreased probabilities of live birth and clinical pregnancy and an increased risk of low birthweight in women with PCOS undergoing their first *in vitro* fertilization embryo transfer cycle (17). In this study, they adjusted for many factors that would affect the results, such as ovarian stimulation starting date, age, BMI, systolic blood pressure, basal testosterone, fasting blood glucose, triglycerides, ovarian stimulation protocol, and number of transferred embryos. Most of the above factors are indicators of metabolic disorders in PCOS. Women with PCOS are at a higher risk of developing metabolic syndrome, and they also have higher levels of inflammatory mediators or gene markers (35, 36). The uterine hyperinflammatory state in women with PCOS might result in maternal pregnancy complications ranging from miscarriage to placental insufficiency (37), which could have significant effects on the reproductive outcomes for women with POCS undergoing IVF procedures. The state of chronic inflammation is not routinely assessed since it is difficult to externalize using conventional indicators. Thus, it is difficult to determine whether elevated serum levels of UA itself or the clinical implications, including chronic inflammation, affect IVF outcomes in women with POCS. From this study, we could not determine whether high serum UA levels would cause unsatisfactory IVF treatment outcomes in non-PCOS patients since they do not share the same metabolic status as PCOS patients. Our study recruited patients without a diagnosis of PCOS. Most of the study population had a normal BMI. We found that serum UA levels had no significant correlation with either clinical treatment outcomes or embryonic treatment outcomes. After adjustment for BMI, the correlation between high serum UA levels and maternal complication rates no longer existed.

There were some limitations of this study. The retrospective nature and data from one single center were limitations. We did not measure the follicular UA level, which might directly influence the microenvironment of follicular development. There were also many strengths of our study. First, the inclusion and exclusion criteria were strict, which ruled out many cofounding factors that would interfere with the results. Second, the embryonic treatment outcomes were thorough, from fertilization to euploid rate, which could assess the potency of oocytes in the most comprehensive way. Third, the study population was patients without PCOS, and the results could be applied to a wider population for pre-IVF intervention consideration.

## Conclusions

This study aimed to explore the association of IVF treatment outcomes and serum UA levels in patients without PCOS. Based on the results of our study, there is no association of clinical significance between serum UA levels and pregnancy outcomes, which means there is no need to adjust serum UA levels to optimize the IVF treatment outcomes. However, the positive correlation between BMI and serum UA levels suggests that lifestyle modification before IVF is still necessary, especially for those for body weight.

## Data availability statement

The original contributions presented in the study are included in the article/supplementary material. Further inquiries can be directed to the corresponding author.

## Ethics statement

The studies involving humans were approved by Independent Ethics Committee for Clinical Research and Animal Trials of the First Affiliated Hospital of Sun Yat-sen University. The studies were conducted in accordance with the local legislation and institutional requirements. The participants provided their written informed consent to participate in this study.

## Author contributions

NY: Conceptualization, Data curation, Formal analysis, Investigation, Methodology, Software, Writing – original draft, Writing – review & editing. JS: Conceptualization, Data curation, Formal analysis, Investigation, Methodology, Resources, Software, Writing – review & editing. HJ: Investigation, Resources, Writing – review & editing. PL: Conceptualization, Data curation, Formal analysis, Methodology, Software, Writing – original draft, Writing – review & editing. SL: Resources, Supervision, Writing – review & editing. YY: Conceptualization, Data curation, Formal analysis, Investigation, Methodology, Project administration, Software, Writing – original draft, Writing – review & editing.
